# Improving yield of a recombinant biologic in a *Brassica* hairy root manufacturing process

**DOI:** 10.1002/bit.28178

**Published:** 2022-08-18

**Authors:** Noemi Gutierrez‐Valdes, Suvi T. Häkkinen, Camille Lemasson, Jonas de Groot, Jean‐Pierre Ele‐Ekouna, Marina Guillet, Florian Cardon, Anneli Ritala

**Affiliations:** ^1^ VTT Technical Research Centre of Finland Ltd. Espoo Finland; ^2^ Samabriva Amiens France; ^3^ BIOPI *Biologie Des Plantes Et Innovation* Amiens France

**Keywords:** alpha‐l‐iduronidase, eGFP, hairy root, plant molecular farming, recombinant protein, secretion

## Abstract

Hairy root systems have proven to be a viable alternative for recombinant protein production. For recalcitrant proteins, maximizing the productivity of hairy root cultures is essential. The aim of this study was to optimize a *Brassica rapa rapa* hairy root process for secretion of alpha‐ l‐iduronidase (IDUA), a biologic of medical value. The process was first optimized with hairy roots expressing eGFP. For the biomass optimization, the highest biomass yields were achieved in modified Gamborg B5 culture medium. For the secretion induction, the optimized secretion media was obtained with additives (1.5 g/l PVP + 1 mg/l 2,4‐ d + 20.5 g/l KNO_3_) resulting in 3.4 fold eGFP secretion when compared to the non‐induced control. These optimized conditions were applied to the IDUA‐expressing hairy root clone, confirming that the highest yields of secreted IDUA occurred when using the defined additive combination. The functionality of the IDUA protein, secreted and intracellular, was confirmed with an enzymatic activity assay. A > 150‐fold increase of the IDUA activity was observed using an optimized secretion medium, compared with a non‐induced medium. We have proven that our *B. rapa rapa* hairy root system can be harnessed to secrete recalcitrant proteins, illustrating the high potential of hairy roots in plant molecular farming.

## Graphical abstract

Hairy root cultures are a promising production platform for biopharmaceuticals. Gutierrez Valdes and co‐workers presented the suitability of Brassica rapa rapa hairy roots for secretion of the biopharmaceutical α‐L‐iduronidase (IDUA). The authors showed that with careful media optimization, the ability of hairy roots to produce and secrete complex proteins was increased. Furthermore, the authors presented data proving the functionality of the secreted IDUA. Thus confirming the high potential Brassica rapa rapa hairy roots hold in future in manufacturing of biologics.

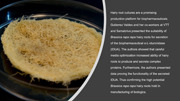

## 1 INTRODUCTION

Over the last three decades, transgenic plants have been established as an alternative system to produce recombinant proteins. At the moment of writing, there are 27 studies for plant‐derived biopharmaceuticals at different stages of clinical trials in the world (US National Library of Medicine, 2022). An example of an already‐in‐the‐market and FDA‐approved plant‐manufactured biopharmaceutical is the recombinant glucocerebrosidase, a therapeutic for Gaucher disease from Protalix BioTherapeutics (Fox, 2012). Currently, the most interesting example of plant biopharmaceuticals is the Health Canada‐approved recombinant coronavirus‐like particle COVID‐19 vaccine from Medicago (Medicago, 2022).

Plant systems for recombinant protein production offer unique advantages such as cost‐effective mass production, absence of inherent human or animal pathogens, and high possibility of glycoengineering. Nevertheless, the associated environmental factors and social acceptance factors limit the implementation of transgenic plant cultivation particularly in open fields (A. M. Lucht, 2015; Shelton et al., 2002). Hairy root cultures integrate both the intrinsic advantages of plant‐based protein synthesis together with production in biocontainment. Hairy root cultures have been widely studied and used for the production of high‐value plant secondary metabolites and recombinant proteins (Cardon et al., 2019; Ele Ekouna et al., 2017a; Häkkinen et al., 2014; Vasilev et al., 2014). Hairy roots also offer genotypic and phenotypic stability and, more importantly, possibility to secrete the expressed proteins (Gutierrez‐Valdes et al., 2020; Halder et al., 2019). Optimizing the protein secretion for a given hairy root culture system, can be advantageous, particularly for the purification of the target proteins (Madeira et al., 2016).

After hairy root culture has been created with molecular farming and synthetic biology tools, the two main approaches for optimization of a hairy root culture system are the biomass production capacity and the target protein secretion. Different kinds and combinations of reagents can be used during the liquid culture. For instance, to ensure better nitrogen availability for the tissue (e.g., using KNO_3_) (Rini Vijayan & Raghu, 2020), to allow wall permeabilization for secretion of target proteins that would otherwise remain bound to the biomass (e.g., using dimethyl sulfoxide; Wongsamuth & Doran, 1997), to protect the integrity of secreted proteins (e.g., using bovine serum albumin [BSA], polyvinyl pyrrolidine [PVP], polyethylene glycol [PEG]) (Alvarez, 2014), or, to minimize the cell lysis by regulating the osmotic pressure in the medium (Ramakrishnan et al., 1999) (e.g., using mannitol; Halder et al., 2019). Statistical modeling can be helpful to design proper experiments that need to include several culture reagents at the same time (Häkkinen et al., 2014, 2018). Ultimately, a well‐rounded optimization should offer synergistic effects of the alternative compounds in the culture to ensure high yields of good‐quality target protein.

The therapeutic alpha‐l‐iduronidase (IDUA), laronidase from Genzyme, is a recombinant form of the human IDUA that is produced by recombinant DNA technology using mammalian Chinese Hamster Ovary (CHO) cell culture (He et al., 2012). The plant‐based analog of IDUA produced in transgenic *Brassica rapa rapa* hairy roots has demonstrated to have reproducible and highly homogeneous glycosylation profiles, as well as similar affinity and specific activity when compared to the one produced by CHO cells (Cardon et al., 2019). IDUA is clinically important as an enzyme replacement pharmaceutical for the treatment of mucopolysaccharidosis type I (MPS I), a progressive lysosomal storage disorder. IDUA (EC 3.2.1.76) is a secreted (71 kDa) lysosomal enzyme that presents a signal peptide (^1^M‐^23^A, released in its final secretion form), and six potential *N*‐glycosylation sites as well as hydroxylation. Optimizing a hairy root system that already consistently produces functional plant‐based IDUA such as the one described by Cardon et al. (2019), represented an opportunity to evaluate if it can be further harnessed to generate higher recombinant protein yields and/or ease the downstream processing.

The aim of this study was to optimize a hairy root process for secretion of IDUA. The process was first optimized with hairy roots expressing green fluorescent protein (eGFP). As a production host we used *B. rapa rapa* hairy roots, which are currently used to develop commercial production bioprocesses and which have shown to possess high recombinant protein production capacity (Huet et al., 2014). Our optimization approach intended to identify a range of culture medium additives that, when used alone or in combination, would increase the productivity of the process, including for usually known as “hard‐to‐produce” recombinant proteins. To evaluate if the eGFP secretion optimized conditions would also result in high secretion of other recombinant proteins, we applied this modified medium to the production of the actual target protein, IDUA, a biologic of medical value harder to produce than eGFP.

## 2 MATERIALS AND METHODS

### 2.1 Establishment of *B. rapa rapa* hairy root lines and their maintenance

The eGFP *B. rapa rapa* hairy root lines were developed by BIOPI (Plant Biology and Innovation) resulting from transformation of the pRD400 vector portraying a double 35S promoter‐SP‐His tag‐eGFP‐CaMV polyA as described by Huet et al. (2014). Similarly, the human IDUA *B. rapa rapa* hairy root line was developed with the same vector but as a gene of interest encoding IDUA, as presented by Cardon et al. (2019).

Fresh hairy roots were subcultured every 3 weeks onto solid modified Gamborg B5 medium containing 10‐fold higher concentrations of copper sulfate and cobalt chloride, and no casein when compared to the original Gamborg B5 (Oksman‐Caldentey et al., 1991) and grown in the dark at +24°C. Before inoculation of liquid media, the hairy roots were subcultured on solid plates for 7 days as a standardized time.

### 2.2 Optimization of induction of eGFP secretion in *B. rapa rapa* hairy root lines

*B. rapa rapa* hairy roots were cultured for 14 days in modified Gamborg B5 medium. Inoculum of 50 mg fresh weight (FW) was taken into 100 ml shake flask with 20 ml of culture medium. Cultivation was performed at + 24°C, 90 rpm (shake radius of 3.2 cm), in the dark. After growth period, the recombinant protein secretion was induced by changing the culture medium and continuing the cultivation for another 14 days. Induction medium consisted of standard amounts of PVP (1.5g/l) and 2,4‐D (1.0 mg/l) with varying concentrations of 1‐naphthaleneacetic acid acid (NAA) (1–20 mg/L), methyl jasmonate (MeJA) (9–250 µM), and potassium nitrate (KNO_3_) (2–35 g/L). Sampling was performed by filtrating the hairy roots under suctions using Miracloth filter, and both hairy roots and culture medium were collected separately, frozen via liquid N_2_ and stored at −20°C. Both, fresh and dry weights (FW and DW) were recorded. For the DW measurement, samples were freeze‐dried over 3 days in −56°C coil temperature CHRIST ALPHA1‐4 LD plus chamber.

### 2.3 Statistical experimental design and statistical analyses

A full factorial design at three levels (Modde v12.0 software) was used to investigate the effects of studied factors on the yield and secretion of the recombinant protein. Studied factors were KNO_3_, NAA, and MeJA. A confidence level of 95% was used for statistical experimental designs.

Statistical analyses were conducted with IBM SPSS Statistics 25 software. Normality of the data was assessed with Shapiro–Wilk's test. Unless otherwise described, the evaluation of one‐way analysis of variance (ANOVA) and Levene test of homogeneity of variances was performed for comparison of means, and significant differences between the samples are determined according to post hoc Tukey's HSD with confidence level *p* < 0.01. With data comprising unequal variances, Dunnett T3 test was used.

### 2.4 Protein analysis

#### 2.4.1 Total soluble protein extraction

Total soluble protein was extracted from freeze‐dried *B. rapa rapa* hairy roots. Hairy root samples of 30 mg (DW) were homogenized with steal beads in a Retsch mill (Retsch MM301, two times 1 min at 20 Hz) both before and after the addition of 1 ml of protein extraction buffer (2% (w/v) sodium ascorbate and 10 mM ethylenediaminetetraacetic acid (EDTA) in phosphate‐buffered saline (PBS) (0.12 M Na_2_HPO_4_·2H_2_0, 0.03 M NaH_2_PO_4_·H_2_O, 1.5 M NaCl, pH 7.4). The insoluble material was removed by centrifugation (Eppendorf Centrifuge 5810 R, 10000 rpm, 10 min, +4°C). Total soluble protein (TSP) content of extracts was measured using the Bradford assay (1976) with Bio‐Rad reagent (Bio‐Rad) and bovine serum albumin (BSA; Sigma‐Aldrich) as standard.

#### 2.4.2 Fluorometric analysis of the eGFG

The eGFP concentration in culture medium and in TSP extracts of *B. rapa rapa* hairy roots was determined by fluorometry. Undiluted spent medium samples coming from different treatments were pipetted in pre‐cooled (+4°C) black microtiter plates (Microfluor 2; Thermo Fisher Scientific) as triplicates. The fluorescence of the samples was determined at 485/527nm using a VICTOR2 plate reader (Perkin Elmer) at 12nm bandwidth and 100 ms measurement time. Sample readings were compared to a standard curve constructed with a purified GFP standard with known concentrations. For analyzing remaining intracellular eGFP after induction, TSP was extracted and analyzed as with the abovementioned fluorometric analysis.

#### 2.4.3 Western blot analysis of IDUA expressing *B. rapa rapa* hairy roots

Western blot analysis of IDUA samples (crude culture media) was performed as previously described (Cardon et al., 2019) with the exception that after the mouse IDUA‐binding primary antibody (ABIN603316 from antibodies‐online), the subsequent secondary antibody used was the IRDye 680RD Goat anti‐mouse (926‐68070 from Li‐cor). Scanning of the blots after incubation and washing was performed with the Odyssey CLx Imaging System. Relative densitometries were analyzed using Image Studio Lite Ver 5.2.

#### 2.4.4 Determination of IDUA activity

Spent medium from the IDUA expressing hairy root line after induction of secretion were analyzed as previously described (Cardon et al., 2019).

### 2.5 Microscopy and photographs of *B. rapa rapa* hairy root lines

*B. rapa rapa* hairy root fragments were excised from the liquid culture, mounted on microscopy slides in water or in toluidine blue, and analyzed with an inverted microscope (IX73 OLYMPUS). Macroscopic pictures were captured using a Nikon D5200 camera.

## 3 RESULTS AND DISCUSSION

### 3.1 Growth behavior of *B. rapa rapa* hairy root line

**Figure 1:**
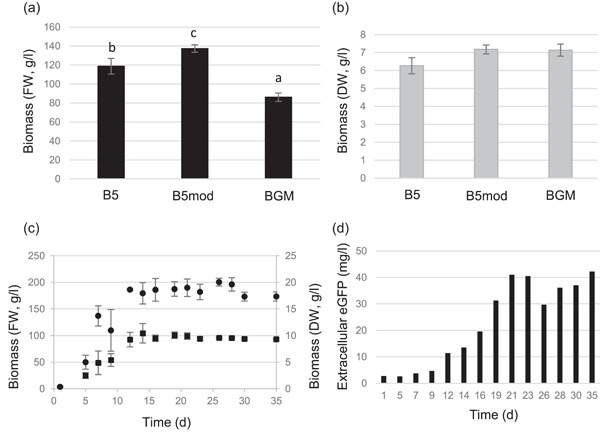
Biomass accumulation, growth behavior, and eGFP secretion of *Brassica rapa rapa* hairy roots carrying eGFP encoding gene. (a) Biomass accumulation in fresh weight (FW, g/L) with B5, B5mod, and BGM after growth period of 14 days. (b) Biomass accumulation in dry weight (DW, g/L) with B5, B5mod, and BGM after growth period of 14 days. (c) Growth behavior on B5mod medium (Circles: biomass expressed in FW; squares: biomass in DW). (d) Extracellular eGFP yield (mg/L) during the cultivation (only one replicate assayed). Error bars represent the standard deviation of four (a and b) or three (c) biological replicates. Letters (a, b, c) indicate the statistically significant differences (*p* < 0.01). BGM, *Brassica* Growth Medium; eGFP, green fluorescent protein

A Gamborg B5 ‐based growth medium for *B. rapa rapa* hairy roots had been previously established by Samabriva (*Brassica* Growth Medium [BGM]). This medium contains less vitamins and microelements, and more sucrose than the usual B5 medium and already allowed a productivity increase of some recombinant proteins. In this study, we assayed whether modified Gamborg B5 ‐based medium (B5mod) could also result in good biomass accumulation with *B. rapa rapa* hairy roots, since earlier it has been successfully used for cultivation of *Solanaceae* hairy roots (Häkkinen et al., 2005, 2014). In addition, the growth in basic Gamborg B5 (B5) medium was studied in parallel (Figure 1a,b). When the accumulation of fresh biomass weight (FW) was evaluated, it was observed that the biomass accumulation in studied media differed statistically (Figure 1a) and the highest biomass was achieved with B5mod media. However, based on dry weight (DW), there were no statistically significant differences in biomass accumulation in the studied media (Figure 1b). Differences in FW and DW results reflected the differences in sucrose concentrations, as BGM medium contained higher sucrose concentration (50 g/L) when compared to the other two (30 g/L). The *B. rapa rapa* hairy roots grown in BGM medium with high sucrose level did not absorb as much intracellular water as hairy roots grown in B5 and B5mod (Figure 1a). Due to the equal growth with lower sucrose level, B5mod was selected as a cultivation medium for biomass production in the rest of the study.

To find out the optimal secretion induction phase, the growth behavior of *B. rapa rapa* hairy root line in B5mod was determined (Figure 1c). An increase in biomass accumulation was observed until Day 14, when stationary stage was reached. Thus, 14 days was selected as the induction point for the test protein, eGFP secretion. An uninduced baseline amount of up to 40 mg/L eGFP was detected in the culture medium already after 21 days when the B5‐mod medium was applied (Figure 1d).

### 3.2 Optimization of induction of eGFP secretion in *B. rapa rapa* hairy roots

Factors to be assessed for secretion induction in *B. rapa rapa* hairy roots were selected according to earlier studies, which have shown to improve recombinant protein secretion in hairy root platforms, including KNO_3_, NAA, and PVP as a stabilizer (Drake et al., 2003, 2009; Häkkinen et al., 2014; Wongsamuth & Doran, 1997). In addition, we wanted to see whether methyl jasmonate (MeJA), a known elicitor for a broad range of plant metabolites, could play a role in recombinant protein secretion. Some studies have reported the use of MeJA to increase the accumulation levels of recombinant proteins (Fraissinet‐Tachet et al., 1998; Karimzadegan et al., 2019). The idea behind using this organic compound as reagent for recombinant protein optimization is that when plants experience herbivore attacks and wounding, plant defense mechanisms get triggered through jasmonate signaling pathways, resulting in reallocation of plant resources to defense mechanisms instead of developmental processes. Hence, energy and resources like amino acids can be shifted to synthesize other proteins such as heterologous proteins.

Secretion induction assays in *B. rapa rapa* hairy roots had shown that addition of 2,4‐D in the culture media was able to increase the extracellular levels of recombinant proteins such as lipase or eGFP (Ele Ekouna et al., 2017). Thus, according to these findings the 2,4‐D level was set to 1mg/L. In addition, a PVP level of 1.5 g/L has been shown to function as a stabilizer of the secreted recombinant protein, which was also set as a standardized level in these experiments (Häkkinen et al., 2014). The studied factors included to the design of experiment (DoE) consisted of KNO_3_, NAA, and MeJA. The optimization iteration was performed in three experimental rounds (Table 1).

**Table 1:** The studied factors and design of experiment (DoE) of *Brassica rapa rapa* hairy root eGFP secretion induction

Factor	First secretion induction	Second secretion induction	Third secretion induction
KNO	5–15 g/L	2–26 g/L	15–35 g/L
NAA	1–20 mg/L	–	–
MeJA	50–250 µM	9–91 µM	10–50 µM
DoE	Full factorial design in two levels	Central Composite Orthogonal (CCO) design with star points	Central Composite Orthogonal (CCO) design with star points
Number of experimental (and central) points	11 (3)	11 (3)	11 (3)

Abbreviation: eGFP, green fluorescent protein.

**Figure 2:**
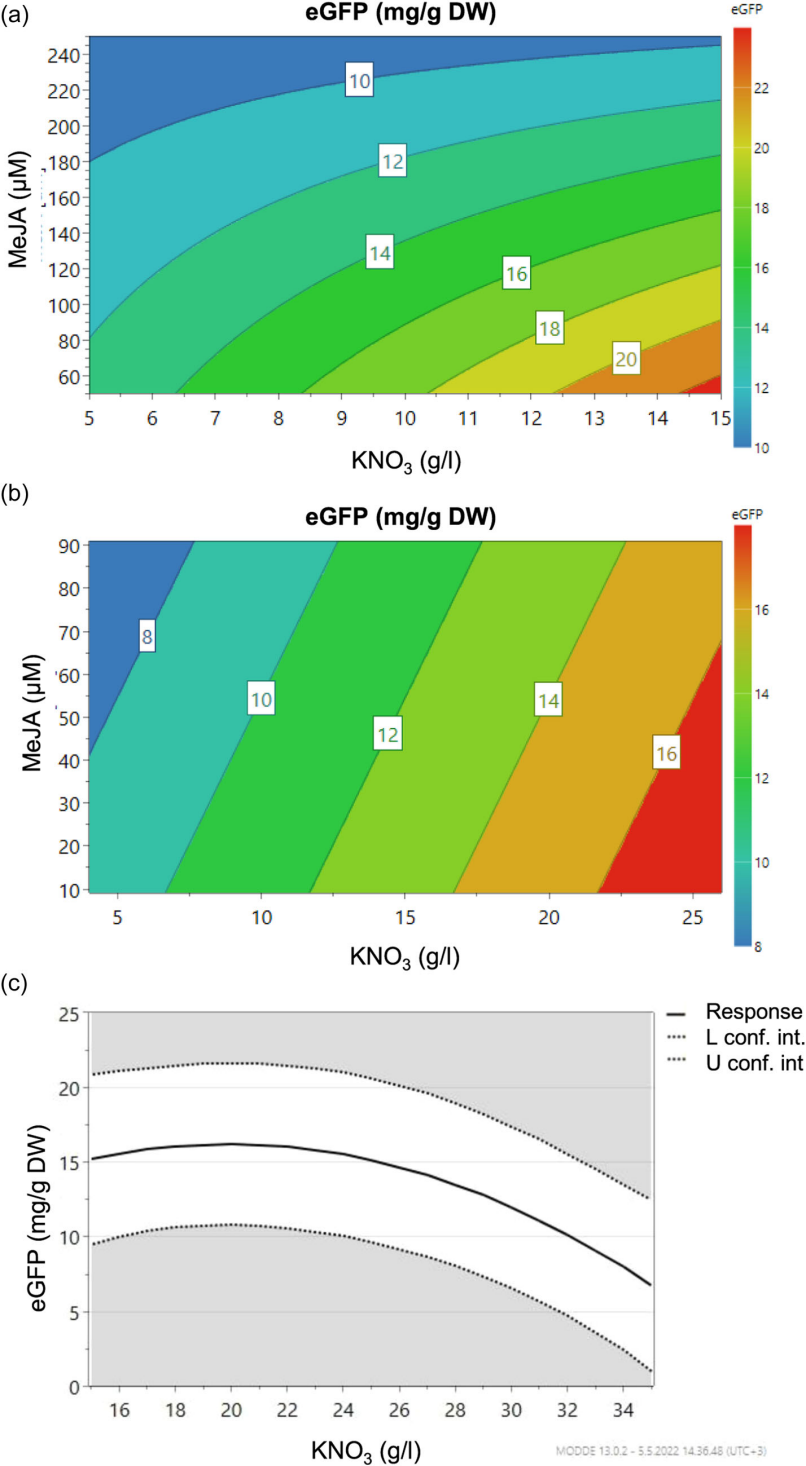
*Brassica rapa rapa* hairy root green fluorescent protein (eGFP) secretion induction experiments. (a) A contour‐plot based on the model constructed from the empirical secretion data of the first secretion induction experiment (Tables 1 and 2) showing secreted eGFP related to MeJA and KNO_3_. (b) A contour‐plot based on the model constructed from the empirical secretion data of the second secretion induction experiment (Tables 1 and 2) showing secreted eGFP related to MeJA and KNO_3_. One outlier (N6) was selected. (c) A contour‐plot based on the model constructed from the empirical secretion data of the third secretion induction experiment (Tables 1 and 2) showing secreted eGFP related to KNO_3_.

The outcome of secretion induction optimization as model descriptors and equations are shown in Table 2. The first secretion induction optimization round indicated that KNO_3_ and MeJA had a significant effect on the eGFP secretion whereas NAA had no influence. The highest eGFP secretion, 22 mg/g DW *B. rapa rapa* hairy root biomass, was obtained in the studied range with low MeJA and high KNO_3_ levels (Figure 2a).

**Table 2:** Descriptions and equations of the models created in *Brassica rapa rapa* hairy root eGFP secretion induction experiments

Model descriptor	First secretion induction	Second secretion induction	Third secretion induction
R^2^	0.930	0.936	0.825
Q^2^	0.852	0.898	0.687
Validity	0.950	0.991	0.782
Reproducibility	0.826	0.789	0.855
Equation	7501.4 + 1228.3[KNO_3_] + 1.93 [MeJA] − 4.47 [KNO_3_] × [MeJA]	7587.7 + 399.4[KNO_3_] − 29.0 [MeJA]	−329.4 + 1.658.1[KNO_3_] − 41.6 [KNO_3_]^2^

Abbreviation: eGFP, green fluorescent protein.

The second secretion optimization experiment was performed so that the optimal secretion point was placed in the center of the experimental range (Table 1). Again, the highest eGFP secretion was gained with high KNO_3_ induction. Noteworthy is that the role of MeJA was observed to be minimal. At highest the eGFP secretion was reaching 16 mg/g DW *B. rapa rapa* hairy root biomass (Figure 2b). Indeed, the insignificant role of MeJA was confirmed in the third secretion induction experiment were only KNO_3_ had a significant effect on eGFP secretion. It was shown that the optimal concentration based on the model would be 20.4 g/L KNO_3_ which would yield 16.2 mg/g DW eGFP (~260mg/L) (Figure 2c). In the study of Häkkinen et al. (2014); the addition of 14 g/l KNO_3_ and 19 mg/L NAA to the B5mod culture medium increased the amount of recombinant M12 antibody recovered 30 fold.

As described above, for *B. rapa rapa* hairy root expressing eGFP, NAA and MeJA did not give additional benefit in secretion induction. Earlier, Drake et al. (2009) showed that the addition of growth regulators (NAA, indolebutyric acid (IBA), 6‐benzylaminopurine (BAP), and kinetin (KIN)) to hydroponic plants all cased an increase in the root biomass, induced the secretion of recombinant proteins and also increased their stability in the hydroponic culture medium. NAA had the greatest effect on rhizosecretion with improving the antibody yields into the liquid media ca. 50‐fold when compared with nonsupplemented hydroponic media. In the current study, auxin was added in the form of 2,4‐D and it is likely that additional auxin supplementation with NAA did not extend the auxin‐induced effect in hairy roots.

**Figure 3:**
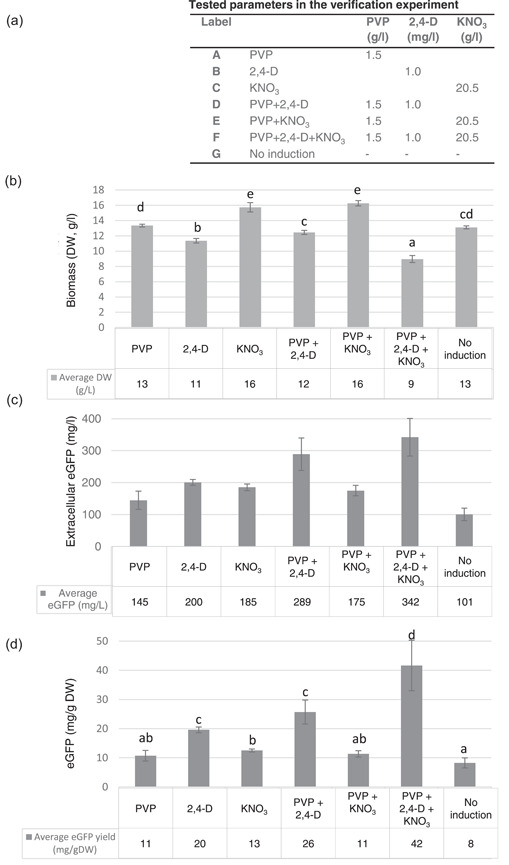
Biomass accumulation and green fluorescent protein (eGFP) secretion in *Brassica rapa rapa* hairy root clone in the secretion induction verification (a) Experimental set‐up, (b) Biomass accumulation (dry weight [DW], g/L) (c) Amount of secreted eGFP to culture medium after induction period (mg/L) (one‐way analysis of variance, post hoc Tukey HSD, *p* < 0.01), (d) Secreted eGFP after secretion induction expressed in mg/g DW hairy root biomass. Error bars represent the standard deviation of five biological replicates. Letters (a, b, c, d) indicate statistically significant differences (*p* < 0.01). PVP, polyvinyl pyrrolidine

To substantiate the identified optimal secretion induction conditions for *B. rapa rapa* hairy roots expressing eGFP, an empirical setup shown in Figure 3a was designed. The biomass accumulation was statistically significantly lower when secretion induction, PVP + 2,4‐D + KNO_3_, was applied as compared to all other treatment and non‐induced samples (Figure 3b). Nevertheless, the PVP + 2,4‐D + KNO_3_ secretion induction resulted in the highest eGFP secretion of 342 mg/L being 3.4‐fold higher than in the noninduced sample that reached level of 101 mg/L (Figure 3c). The highest secretion value corresponded to 42 mg/g DW hairy roots (Figure 3d).

### 3.3 Secretion induction of *B. rapa rapa* hairy root clone expressing IDUA

To understand whether *B. rapa rapa* hairy root clone carrying IDUA expression vector would show similar secretion behavior as eGFP clone, the setup shown in Table 1 was applied. The construct configuration of both clones was the same, for a secretory protein, the only difference was the recombinant protein. Thus, we were evaluating the protein‐dependency in each system by analyzing how each protein was secreted.

**Figure 4:**
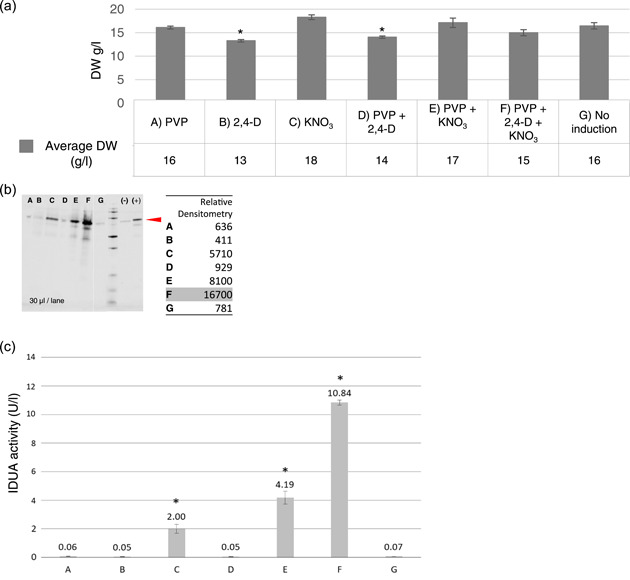
Biomass accumulation and α‐l‐iduronidase (IDUA) secretion in *Brassica rapa rapa* hairy root clone with optimized parameters. (a) Biomass accumulation (dry weight [DW], g/L). Dry weight biomass after secretion induction (Dunnett T3). Asterisks indicate significant differences in levels *p* < 0.05 (*). Error bars represent the standard deviation of four biological replicates (b) Immunodetection of IDUA protein in equivalent amounts of recovered media from the different treatments. A positive control of a commercial standard protein (+) (ABIN1464142, Antibodies‐online) and a negative control (−) of clean non‐induction media. The red arrow shows the 70.8 kDa band corresponding to the mature nonglycosylated standard protein. In the contiguous table, the relative densitometries of the different treatments are displayed. (A = PVP; B = 2,4‐D; C = KNO_3_; D = PVP + 2,4‐D; E = PVP + KNO_3_; F = PVP + 2,4‐D + KNO_3_; G = No induction.] (c) IDUA activity assay on the culture medium of the different treatments applied. Error bars indicate standard deviations. *Indicates significant difference based on Student test (*p* < 0.05). Figure S5 **.** Visual changes in the IDUA‐expressing *Brassica rapa rapa* hairy root clone after the induction of secretion. (A) Color and geotrophic changes in representative bottles of each treatment. (B) Close‐up photographs of representative pieces of hairy roots after the different treatments. Yellow arrows point out swollen tips and hump‐like structures in the treatments with 2,4‐D. The photographs are augmented four times the actual size. (C) Bright‐field microscopic photography of the hump‐like structures resulting from any treatment in which 2,4‐D was used. (D) Bright‐field microscopic photographies from “No induction” and “PVP + 2,4‐D + KNO_3_.” **1** and **2** show the noninduced tissue (water and toluidine blue (TBO), respectively), there is callus/starch (unstained) in some of the tips which might denote possibility of the tissue to keep growing. Blue in the very tips denotes lignified cell walls and the root apical meristem. **3** and **4** show the tissue induced with “PVP + 2, 4‐D + KNO_3_“ (water and TBO, respectively). 2,4‐D induced hump‐like structures can be seen as a proliferation of possibly lateral roots primordia from pericyclic cells. Also, the root‐tip vascular cylinders seem to abruptly deviate due to the formation of the formed humps. Additionally, root tips are swollen and there is absence of root apical meristems (i.e. not blue parts as those in the non‐induced root tips).

In terms of DW biomass, there was a statistically significant reduction of biomass with the samples “2,4‐D” and “PVP + 2,4‐D,” compared with “no induction” (Figure 4a). However, in general terms, the dry weights were in the same range as those for the eGFP clone. This is in line with the general set‐up of the secretion induction experiment, in which the biomass for all treatments was propagated using the same B5mod medium. At that point, all bottles were replicates using the same conditions, and then changing the medium for the different treatments of inducers reflects how these affect the secretion of the respective protein.

The secretion of IDUA protein was analyzed in recovered media from the different treatments after the period of induction. The immunodetection protocol for IDUA detection was performed as described in Section 2.4.3. The “PVP + 2,4‐D + KNO_3_” (treatment F) produced the highest IDUA secretion, as reflected in the highest value for relative densitometry (Figure 4b). Additionally, treatments with “KNO_3_” (treatment C) and with “PVP + KNO3” (treatment E) also induced the secretion of detectable amounts of IDUA protein. In treatments E and F, there are lower bands, indicative of protein degradation. However, we cannot discern the relative activity of protein degradation because of the large absolute differences in overall IDUA protein.

The results of IDUA secretion obtained by immunodetection were confirmed using an enzymatic assay to measure the activity of secreted IDUA in the culture media from the different treatments (Figure 4c). Treatments C, E, and F gave the best results in terms of IDUA activity, especially treatment F with PVP, 2,4D, and KNO_3_ which is higher and statistically different than treatments C and D. Therefore, treatment F allows to obtain the highest amount of active IDUA protein in the studied conditions.

The treatments of induction affected the morphology of the hairy roots (Figure S5). The treatment with “PVP + 2,4‐D + KNO_3_,” for instance, eliminated the tendency of the hairy roots to grow upwards in the flask when compared to the roots with the “no induction” treatment. PVP and KNO_3_, when used independently, did not affect the structure of the tissue when compared to the “no induction” treatment. However, in all treatments in which 2,4‐D was used, the root tips got swollen and they developed hump‐like structures like the ones previously reported by Ele Ekouna (2017) in *B. rapa rapa* 2,4‐d‐treated hairy roots; and by Rage et al. (2020) in *Nicotiana benthamiana* 2,4‐D treated roots. For our experiment, the hump‐like structures were spotted after 4–6 days in treatments supplemented with 2,4‐D. The morphology changes were the same in the case of the eGFP hairy root clone (data not shown).

2,4‐D, affects the morphology of the roots by cell wall remodeling. In presence of a high auxin concentration, the cell wall is loosened, and the turgor pressure against the loosened wall leads to elongation. The general auxin‐induced elongation mechanism has been explained before and, briefly, it states that the auxins activate the H + export, lowering the cell wall pH. This provokes disruption of hydrogen bonding between cellulose microfibrils which in turn loosens the cell wall and consequently elongates cells. After the cell wall elongation, there is a consequent influx of water into the vacuole (Taiz, 1994). For the case of hairy roots, the treatment with 2,4‐D only affects the cell wall expansion in cortex and epidermis cells as suggested in previous studies (Ele Ekouna et al., 2017; Rage et al., 2020). Also, this auxin pressure may have stimulated the formation of lateral roots primordia from pericycle cells. The swelling of roots and of the generated hump‐like structures was a consequence of cell wall remodeling and elongation that in turn provoked uptake of water by those specific newly formed structures.

Nitrogen is a crucial macroelement for protein synthesis (Scheible et al., 2004). As already demonstrated by previous studies (Häkkinen et al., 2014; Holland et al., 2010), culture media supplemented with nitrate improves the intra‐ and extracellular levels of recombinant proteins in tobacco hairy roots and suspension cell cultures, mainly by improving the protein synthesis and stabilizing the secreted proteins. For example, in (Holland et al., 2010) recombinant monoclonal antibody (mAb) accumulation levels were 10‐ to 20‐fold higher and stability of secreted mAb 150‐fold better with increased nitrogen levels. Similarly, in *Nicotiana benthamiana* transient expression system (Fujiuchi et al., 2014) observed that hemagglutinin contents were significantly improved, 40% more, with higher nitrate levels. These findings indicate together with our data that role of elevated nitrogen level can be generalized and is transferrable to other recombinant proteins.

PVP, a water‐soluble polymer, has colloidal and stabilizing properties in different cell cultures while being inert physiologically and metabolically (Magnuson et al., 1996). Some studies have reported that intracellular recombinant proteins were not significantly affected by the addition of PVP, nonetheless, due to the protein stabilizing effect of PVP, the stability of those proteins was significantly improved (Martínez et al., 2005; Pham et al., 2012). On the other hand, in older reports, PVP (up to 3 g/L) is reported to have an effect on organ culture and growth of plant cells (LaCount et al., 1997; Magnuson et al., 1996; Sharp & Doran, 2001).

In an experiment with *Withania somnifera* hairy root culture producing a recombinant globular adiponectin (gAd) as a secretory protein, Dehdashti et al. (2020) showed that their MS media supplemented only with PVP (2 g/L) stabilized by fivefold the extracellular protein being secreted when compared to their control with no PVP added. They also reported that a combination of PVP (1 g/L) and KNO_3_ (2 g/L) resulted in the highest extracellular and intracellular gAd production (1877 μg/L and 21.3 μg/g FW, respectively) significantly higher than their control containing no PVP or KNO_3_. This illustrates that PVP alone can stabilize the protein when it has been secreted to the apoplast, however, it is only with an extra nitrogen source (i.e., KNO_3_) that the overall protein production is boosted and in turn stabilized by the PVP already in the media. The analysis of the secreted IDUA using an enzymatic assay to measure the activity of the protein has demonstrated that the produced recombinant protein was indeed active. This protein in such a production system has been already characterized in terms of glycosylation, homogeneity, and reproducibility (Cardon et al., 2019).

The improvement of the recombinant protein production by hairy roots of *B. rapa rapa* by culture medium optimization gave promising perspectives regarding the industrial production of lysosomal enzymes by a hairy root platform. Indeed, it was already proven that the culture of hairy roots in large‐scale bioreactors for the production of recombinant proteins, such as IDUA, is possible (Cardon et al., 2019; Gutierrez‐Valdes et al., 2020). Hairy root culture in large scale bioreactors for the production of therapeutic compounds is suitable for GMP industrial purpose thanks to a controlled and sterile environment. Moreover, biomass growth and molecule production can be monitored on‐line thanks to identified growth markers (F. Cardon et al. manuscript under preparation) to ensure reproducible batches. The optimized culture medium developed in this study could be applied in large‐scale cultures to improve the production of recombinant protein by hairy roots.

## 4 CONCLUSIONS

Hairy root cultures represent a promising, scalable manufacturing system for high‐value biopharmaceuticals. To maximize the productivity, a careful optimization of the whole production process is required, including optimization of the culture medium for both growth and protein production phases. We studied the suitability of *B. rapa rapa* hairy roots for secretion of a biopharmaceutical, namely, IDUA. Our data first showed that with careful process optimization high level of secretion of a model protein such as eGFP can be obtained. We also demonstrated that such findings can be effectively applied to increase the ability of hairy roots to produce and secrete complex proteins such as IDUA which remains functionally active using the optimized medium. Thus *B. rapa rapa* hairy roots hold high potential as a production platform for complex human glycoproteins such as IDUA and likely also for other biologics.

## AUTHOR CONTRIBUTIONS

Noemi Gutierrez‐Valdes, Suvi T. Häkkinen, Camille Lemasson, and Jonas de Groot conception and design, experimenting, data analysis, interpretation of data, and drafting the manuscript. Jean‐Pierre Ele‐Ekouna providing the eGFP clone, revising the manuscript. Marina Guillet interpretation of data, and revising the manuscript. Florian Cardon and Anneli Ritala conception and design, interpretation of data, drafting, and revising the manuscript. All authors have given final approval for the version to be published.

## CONFLICTS OF INTEREST

Florian Cardon, Camille Lemasson, and Marina Guillet are employed by the company Samabriva SA. The remaining authors declare that the research was conducted in the absence of any commercial or financial relationships that could be construed as a potential conflict of interest.

## Supporting information

Supportinginformation.

## Data Availability

The data that support the findings of this study are available from the corresponding author upon reasonable request.
