# Supplementary material for: Improving yield of a recombinant biologic in a Brassica hairy root manufacturing process
Source: Biotechnol Bioeng. 2022 Aug 18;119(10):2831–41. doi: 10.1002/bit.28178 (PMC9543041; doi:10.1002/bit.28178)
Supplement: Supplementary file 1 — Supportinginformation. [file BIT-119-2831-s001.pdf]

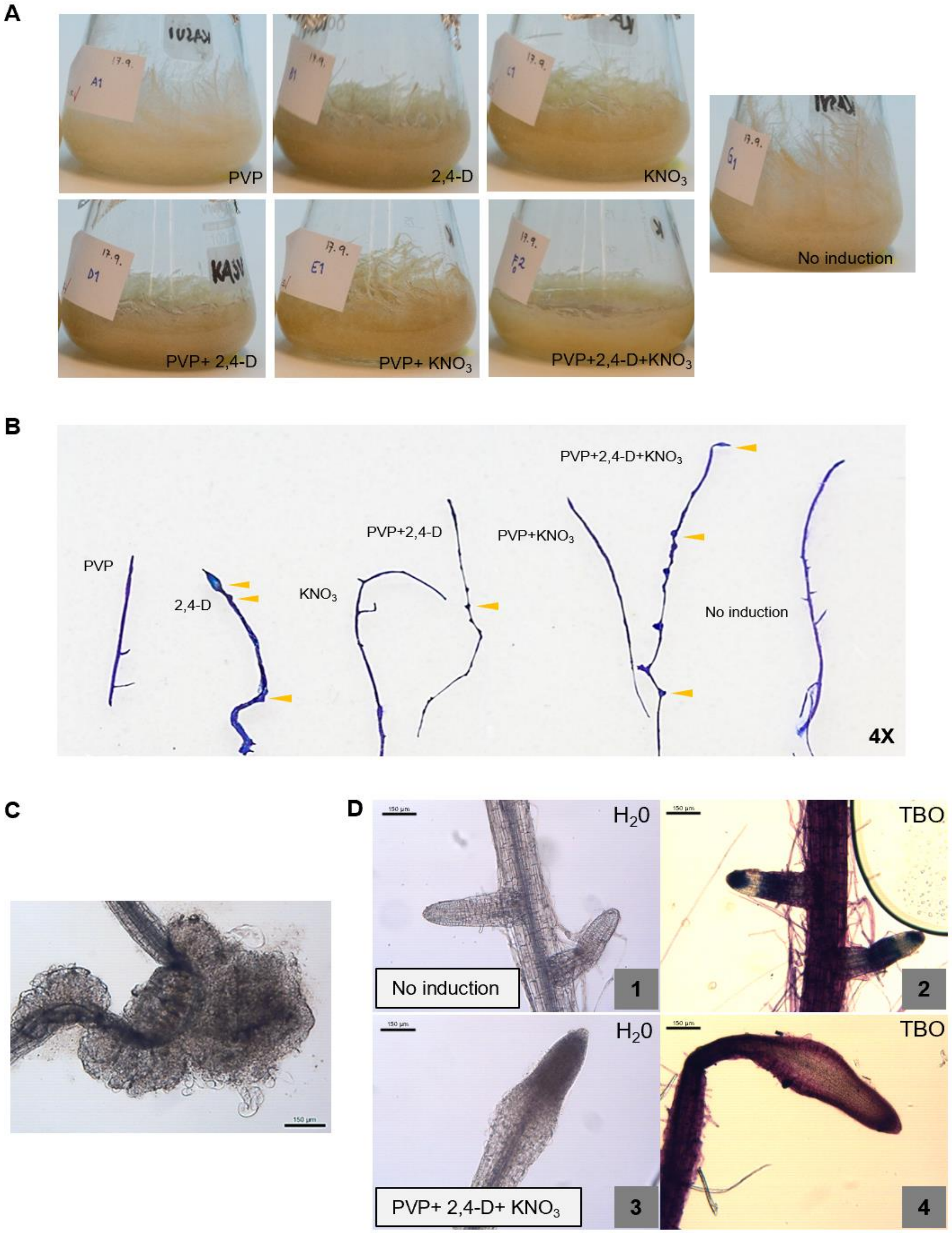

Supporting Figure 5. Visual changes in the IDUA-expressing *Brassica rapa rapa* hairy root clone after the induction of secretion. **A)** Color and geotrophic changes in representative bottles of each treatment. **B)** Close-up photographs of representative pieces of hairy roots after the different treatments. Yellow arrows point out swollen tips and hump-like structures in the treatments with 2,4-D. The photographs are augmented four times the actual size**. C)** Bright field microscopic photography of the hump-like structures resulting from any treatment in which 2,4-D was used. **D)** Bright field microscopic photographies from 'No induction' and 'PVP+2,4-D+KNO_3'. 1 and 2 show the non-induced tissue (water and toluidine blue (TBO), respectively), there is callus/starch (unstained) in some of the tips which might denote possibility of the tissue to keep growing. Blue in the very tips denotes lignified cell walls and the root apical meristem. 3 and 4 show the tissue induced with 'PVP+2, 4-D+KNO_3' (water and TBO, respectively). 2,4-D induced hump-like structures can be seen as a proliferation of possibly lateral roots primordia from pericyclic cells. Also, the root-tip vascular cylinders seem to abruptly deviate due to the formation of the formed humps. Additionally, root tips are swollen and there is absence of root apical meristems (i.e. not blue parts as those in the non-induced root tips).
